# Genito-Urinary Surgery

**Published:** 1895-04-20

**Authors:** 


### Progress in Surgery.

### GENITO-URINARY SURGERY.

(*Continued from page* 30.)

**Stricture of the Urethra.**—Sir W. Maccormac[1] has been taking measurements of the normal urethra, and according to these its average length when unstretched is 6¾ inches. The urethra is usually stated to be eight to nine inches in length. Braune found in a frozen section of a full length man the urethra was only six inches in length. Maccormac considers that if we are able to treat a case of stricture at the very commencement we can cure it by dilatation. This is contrary to the generally received opinion, which may be said to be "once a stricture, always a stricture." Dr. Charles Getz[2] endeavours to prove that nearly all strictures said to be due to gonorrhœa are really caused by a congenital narrowing of the urethra, and that gonorrhœa merely intensifies the obstruction thus caused. He refers to the examination of the urethral canal of new-born infants by Englisch, who found folds either simply of mucous membrane, or with a connective tissue base, in forty-six cases, but whether these folds can be regarded in any way as congenital strictures is very doubtful. Dr. Getz quotes Hunter's saying, "that he doubted very much if stricture commonly, or even ever, arose from gonorrhœa," and also Sir Astley Cooper's who said, "If asked what was the cause of stricture, I should say ninety-nine cases out of every hundred were the result of gonorrhœa." Dr. Getz believes that both a congenital defect and an acquired pathological condition are essential to the formation of stricture.

Mr. Pearce Gould[3] advocates suturing the urethra and perineal wound, layer by layer, after the performance of Wheelhouse's operation for impassable stricture. He considered external urethrotomy was safer than internal, as the surgeon saw exactly what he was doing. Cantalups[4] considers that the wound in internal urethrotomy (which he performs with Maisonneuve's urethrotome) is much less extensive than in forcible dilatation, and the dilatability of the stricture is much greater after urethrotomy. He advises the retention of a catheter after the urethrotomy and drainage with a tube. Morotti[5] strongly recommends rapid dilatation. He passes through the stricture a filiform guide to which a metallic olivary sound of large size is attached, and larger ones are afterwards passed at the same time, and again next day. Then during the next eight to twelve days gradual progressive dilatation is carried on. Buckston Browne[6] advocates internal urethrotomy for strictures very difficult to dilate. He points out that Symes' statement that "external urethrotomy is free from any risk of hæmorrhage or fistulous opening" is not correct, for he has seen and heard of the most intractable hæmorrhage, and has seen and been asked to cure several cases of permanent fistulous opening arising from the operation. He thinks the very worst cases of stricture can be dilated and internally divided at one operation, and the patient need not be detained in bed for longer than a fortnight, and are exposed to no risks either of fistula or hæmorrhage, the results being excellent and lasting.

**Vesical Calculus.**—The advisability of suturing the bladder in cases of supra-pubic lithotomy is raised by two cases recorded by Mr. F. Page, from the Royal Infirmary, Newcastle-upon-Tyne.[7] It is generally held that if the bladder-wall and the urine are healthy, the bladder may be safely sutured, but not otherwise. In Mr. Page's two cases the bladder was not sutured, but the wound in the abdominal wall was united with fishing-gut, with the exception of its lower extremity. The urine was drawn off either by the retention or frequent passage of a catheter. No urine escaped from the wound in either case after the operation. Both patients were adults. In one case the urine was offensive at the time of the operation, in the other it was normal. Mr. Page has on more than one occasion obtained the same result in children.

Dr. George Chismore, of San Francisco, at a meeting of the Association of Genito-Urinary Surgeons, advocated[8] a modification of Bigelow's operation, designed to meet cases in which the prostate is enlarged. Senile hypertrophy of the prostate is usually regarded as a contra-indication to litholapaxy, but Dr. Chismore recommends crushing and evacuation of what can easily be removed, portions of the calculus which fall into "some pouch or lie between the projecting lobes of the prostate" being left for a future operation. In fact, his method is a return to the old plan of incomplete evacuation. He does not use general anæsthesia, but injects one to two fluid ounces of a 4 per cent. solution of cocaine hydrochlorate, which he has never seen produce serious symptoms, and only in a few "mild toxic symptoms, such as nervousness, &c." He operates in his consulting-room. We might expect a rather high mortality, but it is surprising to find that, although he had operated on 52 elderly patients, there had been no death or serious complication. Dr. Watson considered this method of operating a distinctly retrograde one, as the high mortality in former times had been due to the presence of fragments in the bladder. Dr. Otis (of New York) advocated the supra-pubic operation in cases of considerable prostatic enlargement, and suggested that the prostate might be operated on at the same time if deemed advisable.

Perhaps the most important paper on vesical calculus is one by Surgeon-Major Freyer on 852 operations for stone.[9] There was no selection of cases; every patient who came was operated on, "no matter in what condition." Litholapaxy was performed in 598 cases. The disease recurred once in nine instances, and twice in one instance. So long a time had elapsed between the operation and the symptoms of recurrence that Freyer did not consider that it was due to a fragment left behind at the operation; and he says that, so far as his experience goes, recurrence of stone is as frequent after lithotomy as after litholapaxy. Almost all the cases were operated on at a single sitting. Only twice did Freyer intentionally leave some of the calculus to be crushed at a later operation, and in six cases unintentionally were some fragments left behind, which revealed their presence on the following day by the pain and stoppage of urine. As much as possible of the stone was crushed at the first introduction of the lithotrite, so that in most cases of stone of ordinary size only one introduction of the instrument was necessary. During the last few years the operation had been performed in an increasing number of cases without an anæsthetic, or with local anæsthesia by cocaine. Unless enlargement of the prostate was great it was not considered any bar to the performance of the operation, but when very marked supra-pubic cystotomy was performed. Even cystitis with fetid urine was not considered a contra-indication to litholapaxy, as the removal of the stone in such cases—the cause of the cystitis—was usually successful; kidney disease in all its stages was frequently present, and Freyer would not regard this as a contra-indication to the modern method of crushing, or prefer lithotomy in such cases. He crushes stones which most surgeons in this country would remove by supra-pubic cystotomy, and which it would certainly be unwise for men to attempt who are not well practised in crushing small stones. One calculus weighed 6¼ ozs., and was a hard uric acid stone; 308 weighed 3 ozs. and over; and thirty weighed 2 ozs. and over. Cases of encysted calculi have usually been removed by the supra-pubic method, but Freyer crushes them, if possible withdrawing the stone into the general cavity of the bladder and crushing it there, or else in the sac. The occasional difficulty in diagnosis in the case of very small stones is pointed out, and the use of the evacuating bottle and catheter recommended for their detection, the minute calculus being drawn against the end of the catheter by the suction, when the sharp click caused thereby will lead to its detection. Before Freyer began to perform litholapaxy for male children, he had a record of 197 lithotomies, with only one death, so that he had no cause to replace the cutting by the crushing operation on the ground of mortality, and there were many theoretic objections to litholapaxy in children, such as the small bladder, narrow urethra, and liability to rupture of its mucous membrane. However, he has performed litholapaxy now in male children 158 times, with only two deaths, and prefers it on account of the rapidity of cure and the avoidance of a cutting operation, all the objections mentioned above having been proved to be only hypothetical. He has practically abandoned lithotomy both in children and adults. Out of the last 300 cases operated on, in patients of all ages, only in six was lithotomy performed. In the 598 cases in which litholapaxy was performed the mortality was only 1·84 per cent.

**Cystitis and Tubercle of the Bladder.**—Dr. Porter[10] contributes a page on uro-genital tuberculosis in the male. He reports a case in which not only was the bladder, one kidney, the epididymis on both sides, and the prostate affected, but the mucous membrane of the urethra was thickened and hard from the bladder to within one inch of the meatus, and the end of the urethra was ulcerated, and the surrounding tissues hardened, so that the meatus stood open, and so enlarged from erosion that the tip of the index finger could be inserted. The uro-genital disease was associated with phthisis. The author believes that uro-genital tuberculosis most frequently begins in the epididymis or prostate, and is generally secondary The views of various surgeons on the treatment of the disease are referred to. Tuberculosis of the urethra, except as a part of more or less general uro-genital tuberculosis, is considered to be exceedingly rare, and in such cases the deeper urethra only is usually involved, the anterior portion very seldom.

The relation of cystitis to micro-organisms has lately been much discussed. That the organisms are introduced by catheters which are not surgically clean, in the great majority of cases, is generally admitted; but it has been a question whether micro-organisms could gain, or ever did gain, entrance to the bladder so as to set up cystitis by any other means. Posner and Lewin have recently been conducting experiments on the subject,[11] and consider that, under favourable circumstances, intestinal micro-organisms can be taken up by the blood and excreted through the kidneys. They proved this possibility by injecting cultures of the bacillus prodigiosus into the intestine and finding them in the bile, blood, kidneys, and urine. Their experiments did not support the view that bacteria passed directly from the rectum into the bladder. M.M. Boyer and L. Guinard have recently made a communication to the Académié des Sciences[12] on the impermeability of the epithelium of the healthy bladder to poisons. The inflamed or ulcerated mucous membrane does absorb, but not the normal epithelium. They injected toxic doses of strychnine, atropine, morphine, and other alkaloids into the bladder of dogs without producing any symptoms. Control experiments were also made which showed the susceptibility of the animals to the same injections made hypodermically.

**Strangulation of the Testicle.**—In the Progress of Surgery, in The Hospital, January 5th, 1895, p. 243, Laurenstein's paper on strangulation of the testicle by twisting of the spermatic cord is referred to. Johnstone, of Baltimore,[13] records a case. The symptoms were vomiting and pain in the testicle, and the following day crackling on pressure, which was due to emphysema from the gangrene. A hernia was present, and the symptoms suggested the possibility of its strangulation.

**Rupture of the Vas Deferens.**—Mr. Carless[14] reports one of those very rare cases of rupture of the vas deferens. Whilst lifting a piano it slipped, and the whole weight came on the patient, who at once felt a severe pain in the left side of the scrotum and in the testicle, which became swollen and tender. Great pain on sexual intercourse followed, and then some swelling of the epididymis, chiefly of the globus minor. The testicle was removed three years later as some of the symptoms persisted, and the ruptured vas deferens was found quite occluded at one spot.

[1] Clinical Journal, 1894, July 25, p. 207. [2] Medical Record, Nov. 10, 1894, p. 583. [3] Brit. Med. J., Dec. 8, 1894. [4] Brit. Med., Dec. 17, 1894, and Epit. B. Med. Journal, Jan. 12, 1895. [5] Epitome B. Med. Journal, Nov. 3, 1894. [6] Lancet, Dec. 22, 1894. [7] Lancet, Jan. 12, 1895. [8] New York Med. Journal, 1894, Nov. 24, p. 662. [9] Brit. Med. Journal, 1894, Vol. I., 1294. [10] Annals of Surgery, Oct., 1894. [11] Berl. Klin. Woch., Aug. 6, 1894, and Epitome B. M. Journal, Aug. 25, 1894, and Practitioner, Dec., 1894. [12] The Therapeutic Gazette, Oct. 15, 1894. [13] Annals Surgery, May, 1894. [14] Lancet, Sept. 22, 1894.

